# Long non-coding RNAs as monitoring tools and therapeutic targets in breast cancer

**DOI:** 10.1007/s13402-018-0412-6

**Published:** 2018-10-25

**Authors:** Mª Luisa Pecero, Javier Salvador-Bofill, Sonia Molina-Pinelo

**Affiliations:** 1 Unit of Integral Oncology, Instituto de Biomedicina de Sevilla (IBIS) (HUVR, CSIC, Universidad de Sevilla), Seville, Spain; 2 Centro de Investigación Biomédica en Red de Cáncer (CIBERONC), Madrid, Spain; 3 Instituto de Biomedicina de Sevilla (IBIS), Campus Hospital Universitario Virgen del Rocío, Avda. Manuel Siurot, s/n, 41013 Seville, Spain

**Keywords:** LncRNAs, Breast cancer, Chemotherapy, Radiotherapy, Endocrine therapy, Targeted therapy

## Abstract

**Background:**

Current therapeutic strategies that are used to combat breast cancer vary widely and largely depend on its clinicopathological features, including tumor subtype, size, stage, lymph node involvement, the presence of hormone receptors and/or HER2, as well as the degree of proliferative activity. Recent work has focused on improving our knowledge on the molecular mechanisms that underlie this complex disease. Most of the human genome is transcribed into RNAs that do not encode proteins. These noncoding RNAs may act as mediators in the regulation of gene expression. Based on their size and function, noncoding RNAs are classified into small noncoding RNAs (sncRNAs) and long noncoding RNAs (lncRNAs). LncRNAs have been found to play key roles in relevant biological processes, including breast cancer. As such, lncRNAs have been proposed as diagnostic and prognostic biomarkers, as predictive biomarkers and as putative therapeutic targets.

**Conclusions:**

In this review, we discuss the potential application of lncRNAs for the monitoring and treatment of breast cancer. We conclude that lncRNAs play important roles in the pathophysiology of this disease and may serve as putative therapeutic targets. As such, tumor-specific lncRNAs may be instrumental for improving current breast cancer clinical practices.

## Introduction

Breast cancer is the most frequent malignancy in women and the second leading cause of cancer death in women worldwide [1]. Despite recent advances that have been made in early detection and therapeutic intervention, approximately 30%–40% of patients with localized disease will develop relapses and/or metastases [2–4]. Breast cancer is heterogeneous in nature and encompasses multiple intrinsic tumor subtypes. Based on recently developed gene expression profiling techniques, a classification into four major subtypes has been established with prognostic and therapeutic relevance [5–7]: (1) the luminal A subtype, which represents >50% of the breast cancer diagnoses and is characterized by high estrogen receptor (ER) and progesterone receptor (PR) expression levels and a lack of human growth factor receptor type 2 (HER2) expression (Table 1). These tumors exhibit a low proliferation rate (Ki-67 positivity in <14% of the cells) and a relatively good prognosis due to a high response rate to hormone therapy [8], (2) the luminal B subtype, which represents ~20% of the breast cancer diagnoses. Although these tumors are characterized by high hormone receptor expression levels and a lack of HER2 expression, they exhibit a high proliferation rate (Ki-67 positivity in ≥14% of the cells), a worse prognosis and a worse response to hormone therapy compared to the luminal A subtype. Among the luminal B tumors that have acquired resistance to hormone therapy, ~10% resembles the triple-negative subtype (i.e., ER-, PR-, HER2-; see below) in their sensitivity to chemotherapy [9], (3) the HER2 subtype, which comprises 15–20% of the breast cancers and is characterized by overexpression of HER2 and, in 50% of the cases, the expression of hormone receptors (ER and/or PR). This subtype has a worse prognosis than the luminal subtypes and is relatively resistant to chemotherapeutic agents and tamoxifen [10]. The prognosis of this subgroup has recently been modified [11] due to the development of an anti-HER2 monoclonal antibody, trastuzumab (Herceptin), and other specific treatment regimens, and (4) the triple-negative breast cancer (TNBC) subtype, which represents 15–20% of the breast cancer diagnoses and is characterized by a complete lack of hormone receptor and HER2 expression, which limits the treatment options and renders the tumors highly aggressive. Approximately 40% of the patients with this latter subtype responds well to initial chemotherapy, whereas the remaining patients show a poor response and, concomitantly, a worse prognosis. In addition, patients who carry mutations in either the *BRCA1* or *BRCA2* genes are at a high risk of developing metastases [12]. These are usually TNBC patients. TNBC is the most heterogeneous subtype and comprises, based on molecular profiling, six distinct subtypes, i.e., basal (BL1 and BL2), immunomodulator (IM), mensenchymal (M), mesenchymal stem cell-like (MSL) and luminal types expressing androgen receptors (LARs) [13].

**Table 1:** Classification of breast cancer subtypes and their current treatment modalities

Subtype	Inmunohistochemical characteristics	Treatment
Luminal A	ER+/PR+/HER2-	• Endocrine therapy
		• Chemotherapy
		• Radiotherapy
Luminal B	ER+/PR+/HER2-	• Endocrine therapy
		• Chemotherapy
		• Radiotherapy
HER2	ER-/PR-/HER2+ or ER+/PR+/HER2+	• HER2-targeted therapies
		• Endocrine therapy
		• Chemotherapy
		• Radiotherapy
Triple Negative (TNBC)	ER-/PR-/HER2-	• Chemotherapy
		• Radiotherapy

*ER* estrogen receptor, *PR* progesterone receptor, *HER2* human epidermal growth factor receptor 2

Due to the observed heterogeneities, the options to treat breast cancer are variable and largely depend on the clinicopathological features of the tumors at the time of diagnosis, including tumor subtype, size, stage, lymph node involvement, the presence of hormone receptors and/or HER2, as well as the degree of proliferative activity [14]. According to these variables, the patients will receive neoadjuvant treatment followed by surgery or, conversely, surgery followed by adjuvant treatment. Neoadjuvant treatment is used to reduce locally advanced disease and/or large tumors before surgical removal. An improvement in pathologic complete response (pCR) is commonly used as a surrogate marker for disease-free survival (DFS) and overall survival (OS). For the luminal A and B subtypes, neoadjuvant treatment includes the administration of endocrine therapeutic agents [15] such as estrogen receptor antagonists (tamoxifen), estrogen receptor expression modulators (fulvestrant) or inhibitors of the synthesis of these receptors (letrozole, anastrozole, exemestane), sequentially with chemotherapy when required. For the HER2-positive tumors, neoadjuvant treatment is based on the administration of monoclonal antibodies (trastuzumab, pertuzumab) that block the activity of HER2, in combination with chemotherapy [16]. In case of hormone receptor positivity, neoadjuvant treatment is used sequentially with endocrine therapy. For TNBCs the current conventional neoadjuvant treatment includes sequential chemotherapy based on anthracyclines, followed by taxanes [17]. For breast cancer patients carrying a *BRCA* mutation, the use of cisplatin as monotherapy has been found to be highly effective, resulting in pCRs of 72–83% [18]. Adjuvant treatment is commonly used with the intention to reduce the rates of loco-regional relapses as well as the appearance of distant metastases. In the luminal A and B subtypes of breast cancer, hormone therapy has been found to improve survival rates, and it has been proposed that it should be administered during at least 5 years after surgery [19]. For HER2-positive tumors, adjuvant treatment with trastuzumab is recommended, usually in combination with taxanes [20]. In case these tumors also express hormone receptors, endocrine therapy is added to the drug combination. For patients with TNBC conventional adjuvant chemotherapy has so far remained the golden standard [21].

## LncRNAs and breast cancer

It is well established now that < 2% of the total human genome transcribes RNAs that encode proteins, whereas most of the transcribed RNAs do not encode proteins. During the last decade, it has been reported that these so-called noncoding RNAs may act as mediators in the regulation of gene expression [22–26]. Based on their size and function, noncoding RNAs are classified into small noncoding RNAs (sncRNAs) and long noncoding RNAs (lncRNAs). SncRNAs are smaller than 200 nucleotides and can be subclassified into different groups according to their function, including microRNAs (miRNAs), piwi interacting RNAs (piRNAs), ribosomal RNAs (rRNAs), small interfering RNAs (siRNAs), small nuclear RNAs (snRNAs), small nucleolar RNAs (snoRNAs) and small circular RNAs (cRNAs) [27, 28]. Of these sncRNAs, the group of microRNAs is most elaborately characterized, including its biogenesis, normal function and involvement in disease [29]. The lncRNAs represent a heterogeneous group of RNAs that may function either as primary or as spliced transcripts. These RNAs can be subclassified into five categories: sense, antisense, bidirectional, intronic and intergenic [30]. LncRNAs are usually transcribed by RNA polymerase II and may undergo post-transcriptional modifications in a similar manner as mRNAs [31]. LncRNAs may be localized in the nucleus or in the cytoplasm or, occasionally, in both. In the nucleus, these RNAs may participate in chromatin remodeling, transcription regulation and RNA processing, whereas in the cytoplasm they usually exert their functions through interactions with mRNAs and proteins [32, 33].

In recent years, research on lncRNAs has increased significantly since it has been found that they may play key roles in relevant biological processes, in particular the regulation of gene expression through epigenetic and non-epigenetic mechanisms under both normal and pathologic conditions, including cancer [34]. It has amply been reported now that lncRNAs may promote various tumor suppressing and tumor promoting pathways. As such, they appear to represent novel paradigms for malignant transformation and progression. In breast cancer, several lncRNAs have been identified that exhibit expression patterns different from those in non-tumorous breast tissues [35, 36]. Yang et al. [37] found, for example, that more than 1300 lncRNAs were differentially expressed in HER2-positive breast cancers. Similarly, Shen et al. [38] found that 1758 lncRNAs were deregulated in breast cancers with a triple-negative phenotype compared to paired normal tissues. These results underscore a relevant role of lncRNAs in the development of breast cancer. In addition, it has been found that lncRNAs may be expressed either ubiquitously or in a tissue-specific manner [39] and that they may be released in a stable form into the bloodstream during the course of the disease. Accordingly, Liu et al. [40] recently observed a good correlation between the levels of three lncRNAs (ANRIL, HIF1A-AS2 and UCA1) in plasma and tumor tissues of patients with TNBC. Additionally, our group recently identified three circulating lncRNAs (GAS5, ZFAS1 and RMRP) that were found to be deregulated in patients with advanced breast cancer [41]. As such, these lncRNAs may serve as novel noninvasive biomarkers.

LncRNA expression profiles may also differ in different histological breast cancer subtypes. These lncRNAs have the potential to be used as diagnostic biomarkers and/or as therapeutic targets [42]. In recent years, several lncRNAs have been identified exhibiting oncogenic roles in breast cancer, including H19, HOTAIR, MALAT-1, CCAT1, CCAT2 and UCA1 [43–48]. LncRNAs that were found to exhibit tumor suppressor roles include GAS5, EPB41L4A-AS2, BC040587 and FGF14-AS2 [49–52]. These lncRNAs have been proposed to serve, not only as diagnostic/prognostic biomarkers, but also as predictive biomarkers and as putative therapeutic targets in breast cancer (Table 2) [53].

**Table 2:** Breast cancer-related lncRNAs

LncRNAs	Gene ID	HGNC ID	Expression	Tumor	Function	Key factors	Ref.
ANRIL	100,048,912	34,341	upregulated	triple-negative breast cancer	diagnostic biomarker		[40]
HIF1A-AS2	100,750,247	43,015					
UCA1	652,995	37,126					
GAS5	60,674	16,355	downregulated	advanced tumors	progression biomarkers		[41]
ZFAS1	441,951	33,101					
RMRP	6023	10,031	upregulated	advanced luminal and Her2+ tumors	progression biomarker		[41]
H19	283,120	4713	upregulated	primary breast cancer	Tumor growth	c-Myc	[43]
HOTAIR	100,124,700	33,510	upregulated	breast cancer stem cells	poor prognosis, metastasis, invasion, and short overall survival	STAT3 pathway	[44]
						miR-7	
MALAT-1	378,938	29,665	upregulated	primary breast cancer	prognostic biomarker	YAP	[45]
						PI3K-AKT pathway	
CCAT1	100,507,056	45,128	upregulated	primary breast cancer	prognostic biomarker		[46]
CCAT2	101,805,488	47,044	upregulated	MDA-MB-231 and MCF-7 cells and primary breast cancer	tumor growth	Wnt signaling pathway	[47]
UCA1	652,995	37,126	upregulated	MCF-7 and MDA-MB-231 breast cancer cells	tumor growth	p27 (Kip1)	[48]
GAS5	60,674	16,355	downregulated	breast cancer cells	apoptosis		[49]
EPB41L4A-AS2	54,508	25,643	downregulated	primary breast cancer	inhibition of tumor proliferation, prognostic biomarker		[50]
BC040587	100,506,724	40,352	downregulated	primary breast cancer	predictive biomarker		[51]
FGF14-AS2	283,481	44,368	downregulated	primary breast cancer	progression and prognosis biomarker		[52]
CRALA	80,069	21,598	upregulated	primary breast cancer	chemoresistance and prognosis biomarker		[57]
ARA	5063	8592	upregulated	MCF-7 breast cancer cells	adriamycin resistance	G2/M arrest	[58]
H19	283,120	4713	upregulated	ER+ breast cancer cell lines	chemoresistance	BIK	[64]
CCAT2	101,805,488	47,044	upregulated	lymph node positive breast cancer	resistance to cyclophosphamide/methotrexate/fluorouracil-based chemotherapy		[65]
HOTAIR	100,124,700	33,510	upregulated	MDA-MB231 breast cancer cells	radioresistance	PI3K/AKT-BAD pathway, HOXD10	[70]
UCA1	652,995	37,126	upregulated	LLC2, LCC9, MCF-7-R and T47D-R breast cancer cells	tamoxifen resistance	mTOR signaling pathway	[72–74]
						Wnt-β-Catenin signaling	
						miR-18a/HIF1α feedback loop	
HOTAIR	100,124,700	33,510	upregulated	primary breast cancer tissues and MCF7 breast cancer cells	tamoxifen resistance	GREB1	[76]
BCAR4	400,500	22,170	upregulated	ER+ primary breast cancer	tamoxifen resistance	HER2 signaling pathway	[78]
CCAT2	101,805,488	47,044	upregulated	breast cancer cells	tamoxifen resistance	ERK/MAPK signaling pathway	[82]
UCA1	652,995	37,126	upregulated	breast cancer cells	trastuzumab resistance	miR-18/YAP1	[85]
GAS5	60,674	16,355	downregulated	SKBR-3/Tr breast cancer cells and primary breast cancer tissues	trastuzumab resistance and prognosis biomarker	PTEN/miR-21	[87]
ATB, alias AL589182.3	unassigned	unassigned	upregulated	SKBR-3 breast cancer cells and primary breast cancer tissues	trastuzumab resistance	TGF- β	[89]
						miR-200c	
						ZEB1 and ZNF217	

## LncRNAs as predictive biomarkers in breast cancer

**Fig. 1:**
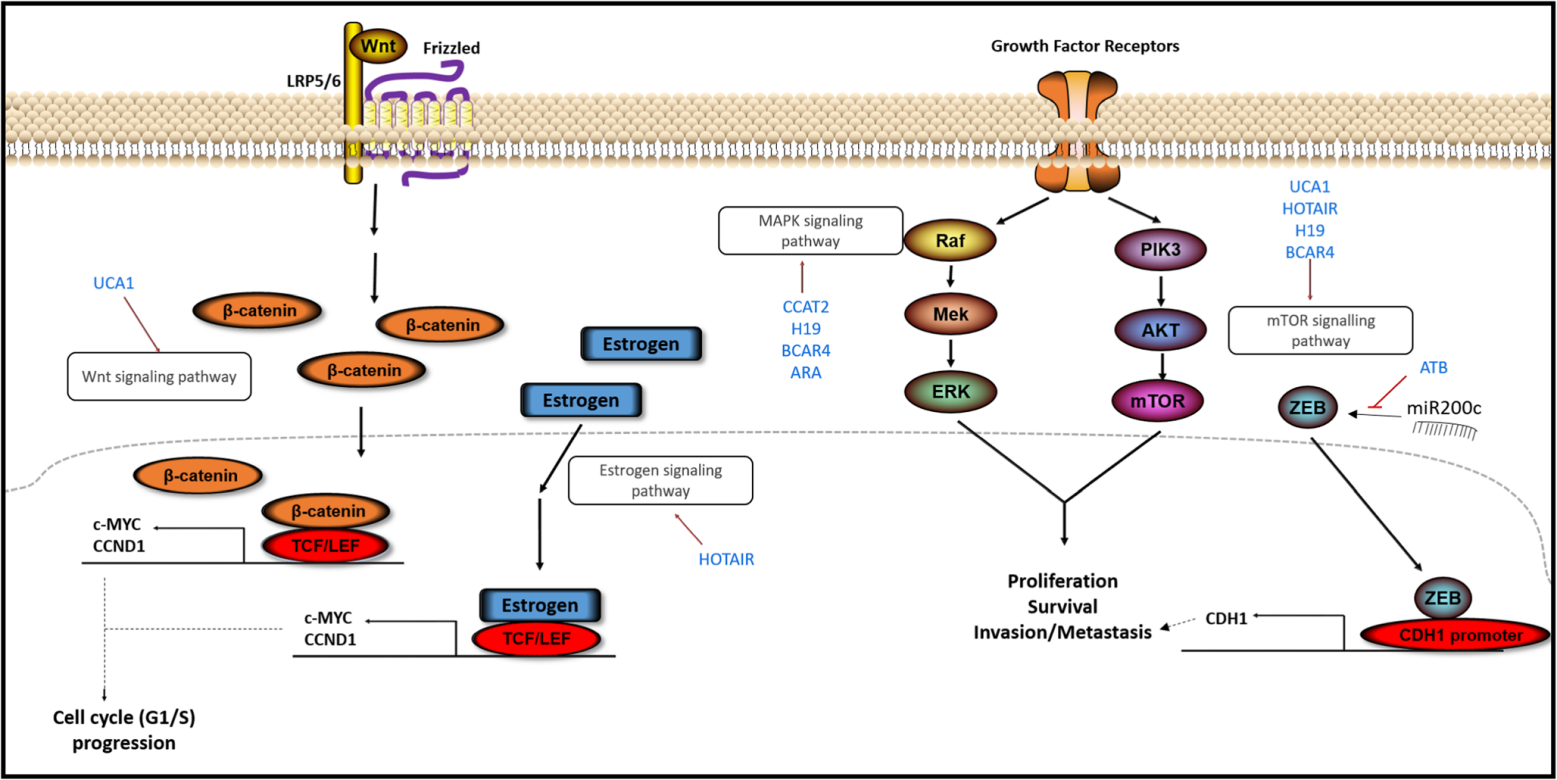
Implications of lncRNAs in signaling pathways in breast cancer

**Fig. 2:**
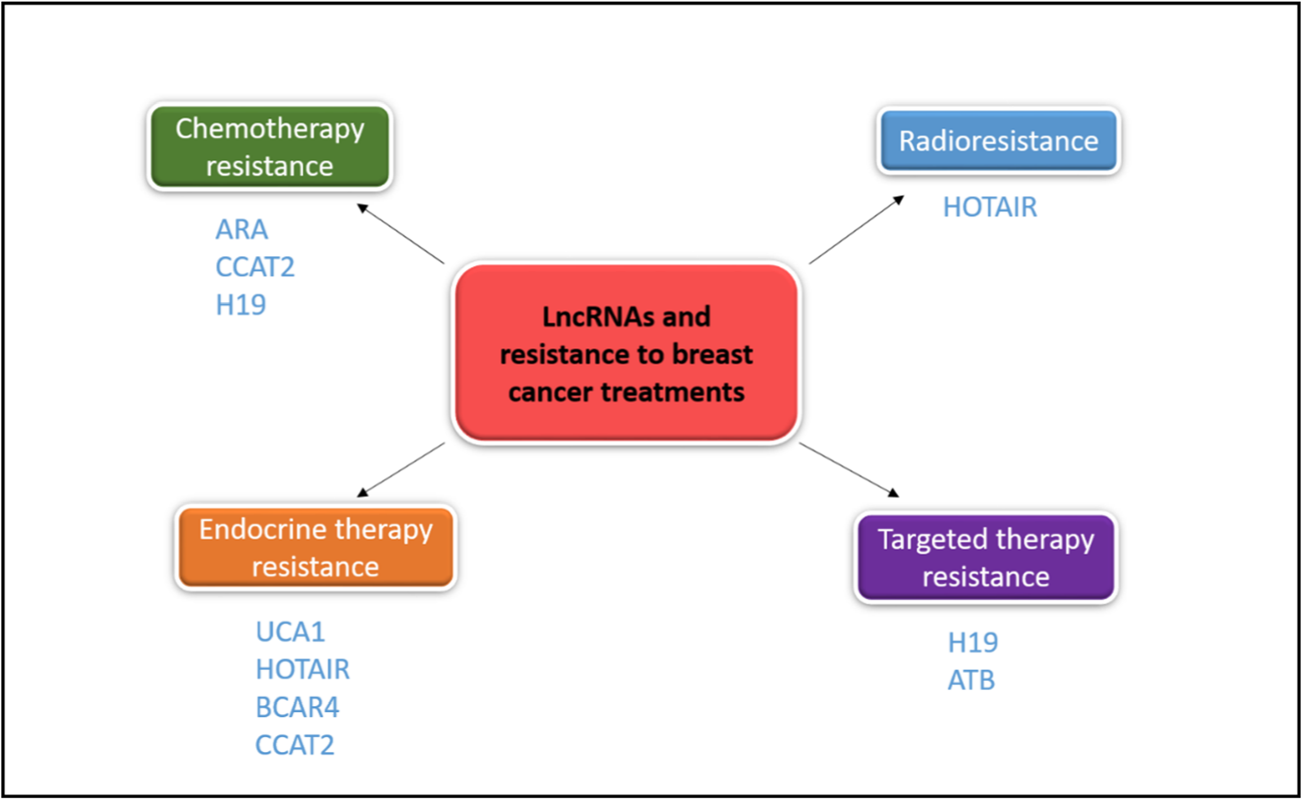
Overexpressed lncRNAs involved in resistance to breast cancer treatment

Although several therapeutic approaches have been developed for breast cancer that have improved the course of the disease, the relapse rates remain high, mainly due to the acquisition of therapy resistance, which is independent of the breast cancer subtype and the treatment regimen used [1]. It has been found that lncRNAs may play crucial roles, not only in the activation of oncogenic signaling pathways that lead to malignant transformation, but also in the resistance of breast cancer to therapy through diverse mechanisms (Fig. 1) (Table 2). Here, we will discuss lncRNAs that have been identified to date as being implicated in the development of resistance to the principal breast cancer treatment regimens (Fig. 2).

### LncRNAs and chemotherapy

Chemotherapy is the most common treatment option used (see above) and consists of the administration of anthracyclines and/or taxanes, although in selected patients cyclophosphamide, methotrexate and/or 5-fluorouracil may be used [54]. Since tumors may develop resistance to these agents, there is a growing interest in the identification of chemotherapy resistance biomarkers [55]. Several LncRNAs have been found to play a role in chemotherapy resistance in several tumors. Fan et al. [56] found, for instance, that in patients with metastatic bladder cancer after cisplatin treatment lncRNA UCA1 was overexpressed. In addition, using in vitro assays, they found that UCA1 increased cisplatin resistance of bladder cancer cells through the Wnt signaling pathway. Another lncRNA, CRALA, has been found to be associated with a poor prognosis in breast cancer patients under a neoadjuvant chemotherapy treatment regimen. This lncRNA was also found to be overexpressed in vitro in breast cancer cells resistant to chemotherapy [57]. LncRNA ARA has been implicated in multiple signaling pathways involved in breast cancer development, including mitogen-activated protein kinase (MAPK) and cell adhesion-related signaling pathways, as well as pathways regulating cell cycle progression. Jiang et al. [58] carried out lncRNA transcriptome profiling in adriamycin-resistant breast cancer cells and found that ARA may be responsible for this resistance. These profiling results were subsequently corroborated by in vitro assays using MCF-7 and MDA-MB-231 breast cancer-derived cells. The authors found that ARA expression knockdown resulted in drug-resistance reversion, as well as in inhibition of cellular proliferation and migration and in promotion of apoptosis and G2/M arrest in adriamycin-resistant cells. LncRNA H19 has been found to be highly expressed in most human cancers, including breast cancer [59–62] and to be correlated with a poor prognosis [63]. Si et al. [64] found that H19 may confer chemoresistance to ER-positive breast cancer cells through silencing the pro-apoptotic gene *BIK*. The authors found that the H19 expression levels increased concomitantly with increasing resistance levels to paclitaxel in different ER-positive breast cancer-derived cell lines. When a resistant cell line was ER negative, however, this effect did not occur. The authors also found that the H19-mediated resistance was P-glycoprotein dependent. The H19-mediated silencing of the *BIK* gene was found to be brought about by EZH2 recruitment and subsequent histone H3 trimethylation (H3K27). The authors suggested that anti-ER-H19-BIK therapies may be used to increase the clinical efficacy of breast cancer chemotherapy. Redis et al. [65] found that lncRNA CCAT2 was overexpressed in breast cancer patients with lymph node-positive disease who received adjuvant cyclophosphamide/methotrexate/5-fluorouracil-based therapy. The authors suggested that CCAT2 may serve as a prognostic biomarker for breast cancer patients with lymph node-positive disease, predicting metastasis and a poor survival.

### LncRNAs and radiotherapy

Radiation therapy is the method of choice for the treatment of inoperable, locally advanced breast tumors. As for chemotherapy, however, also radio-resistance may be acquired during treatment. The underlying biological mechanisms may provide explanations for the variability observed in patient responses. In recent years, the putative role of lncRNAs in the acquisition of radio-resistance in different solid tumors has been assessed by different investigators. Zhou J. et al. [66], for instance, carried out a microarray-based expression analysis in a hypopharyngeal squamous cell carcinoma (HSCC)-derived radio-resistant cell line, RS-FaDu, at 0, 2 and 48 h after radiation exposure. In doing so, they found two upregulated lncRNAs (TCONS_00010875 and TCONS_00018436) and two downregulated lncRNAs (ENST00000470135 and hox-HOXD10–35) in the radio-resistant cell line. Among these four lncRNAs, TCONS_00018436 was considered most promising due to its implication in radio-resistance in an in vitro assay. On the other hand, Hu et al. [67] found that low expression levels of lncRNA ANRIL inhibited proliferation, induced apoptosis, and enhanced radio-sensitivity in nasopharyngeal carcinoma (NPC)-derived cells through miR-125a regulation. Along the same line, Lu et al. [68] found that lncRNA NEAT1 may regulate epithelial-mesenchymal transition (EMT) and radio-resistance by modulating the miR-204/ZEB1 pathway in primary NPC tissues and NPC-derived cells, whereas Jin et al. [69] found that lncRNA MALAT1 may regulate cancer stem cell (CSC) activity and resistance to radiotherapy by modulating the miR-1/Slug pathway in NPC cells, both in vitro and in vivo. It has also been reported that MALAT1 may modulate the radio-sensitivity of high-risk human papillomavirus-positive cervical cancers via ‘sponging’ miR-145.

Although few studies have addressed the role of lncRNAs in radio-sensitivity of inoperable, locally advanced breast tumors, this shortage does not negate the possibility that lncRNAs may be relevant. Recently, Zhou et al. [70] investigated the radio-sensitizing effects of lncRNA HOTAIR on breast cancer cells and its underlying mechanisms. To this end, they analyzed the expression of HOTAIR in five breast cancer-derived cell lines and in one non-tumorous breast cell line. They found that the HOTAIR expression level was lower in one tumor cell line (MDA-MB231) compared to the non-tumorous control cell line. After exogenous overexpression of HOTAIR in the MDA-MB231 cell line it was found that this overexpression had a significant positive effect on its proliferation, which was maintained even after irradiation, suggesting that HOTAIR may play a role in radio-resistance and, as such, may serve as a suitable biomarker. In addition, it was found that the development of radio-resistance by the HOTAIR overexpressing cells was mediated by the PI3K/AKT-BAD pathway and by HOXD10. This lncRNA has also been found to be involved in the radio-sensitivity of colorectal cancer (CRC) cells and Yang et al. [71] reported that HOTAIR silencing enhanced the inhibitory response to irradiation in CRC cells.

In summary, several lncRNAs related to radio-resistance of breast cancer cells have been identified. Since radiotherapy is currently the only therapeutic option for at least some tumor types, additional in-depth analyses are warranted.

### LncRNAs and endocrine therapy

Endocrine therapy, also called hormone therapy, is indicated in patients with detectable estrogen receptor (ER) expression levels, irrespective the use of chemotherapy and/or targeted therapy. In pre-menopausal patients, the standard treatment is tamoxifen for 5–10 years, whereas in post-menopausal women aromatase inhibitors and tamoxifen are valid options [54]. However, tamoxifen resistance limits the long-term effects of the treatment of ER-positive breast cancers. Based on these premises, there is a growing interest in uncovering the molecular mechanisms underlying tamoxifen resistance and to identify suitable prognostic and/or predictive biomarkers. Several lncRNAs have been reported to play relevant roles in the modulation of therapy responses in breast cancer cells. It has, for example, been found that lncRNA UCA1 may confer tamoxifen resistance by activating the mTOR signaling pathway. Wu et al. [72] found that UCA1 expression knockdown in tamoxifen-resistant MCF7-derived LCC2 and LCC9 breast cancer cells increased their apoptotic rates upon endocrine treatment, and this increase was accompanied by a significant reduction in activation of the AKT and mTOR proteins. Conversely, it was found that exogenous overexpression of UCA1 in tamoxifen-sensitive MCF-7 cells decreased the apoptosis induced by hormone therapy, which could be reverted by the mTOR-specific inhibitor rapamycin. Liu et al. [73] found that UCA1 induces tamoxifen resistance by increasing the activity of Wnt/β-Catenin signaling. These researchers observed higher UCA1 expression levels in primary breast cancer tissues compared to normal breast tissues. In addition, they observed a positive correlation between breast cancer stage and UCA1 expression. The authors also reported that the expression of UCA1 was dramatically increased in tamoxifen-resistant breast cancer-derived cells, MCF-7-R and T47D-R, compared to the respective parental cells. Subsequent UCA1 overexpression in parental cells and UCA1 expression knockdown in its derived tamoxifen-resistant cells confirmed the contribution of this lncRNA to this resistance. They also showed that UCA1 expression knockdown increased tamoxifen sensitivity and promoted apoptosis. Furthermore, the authors confirmed the effect of UCA1 expression knockdown on the in vivo tumorigenicity of MCF-7-R and T47D-R cells using a nude mouse xenograft model. In doing so, they confirmed that the mechanism underlying the modulation of tamoxifen resistance by UCA1 was mediated by the Wnt/β-catenin signaling pathway. It has also been shown that UCA1 may increase tamoxifen resistance through a miR-18a/HIF1α feedback loop [74]. Moreover, Xu et al. [75] showed that exosome-borne UCA1 may mediate the transfer of tamoxifen resistance to breast cancer cells. The authors also found that the exosomal UCA1 levels of resistant cells were approximately 27-fold higher than those of tamoxifen-sensitive cells. Additionally, they found that UCA1 expression knockdown in tamoxifen-resistant cells significantly decreased their exosomal UCA1 levels. Accordingly, it was found that exosomes isolated from resistant cells could modulate the effect of endocrine therapy in sensitive cells, which resulted in an enhanced cell viability and a decreased caspase-3 expression level. Accordingly, it was found that UCA1-defective exosomes were unable to induce tamoxifen resistance in sensitive cells.

Xue X. et al. [76] found that lncRNA HOTAIR may increase ER signaling and confer tamoxifen resistance to breast cancer cells. They showed that HOTAIR expression was significantly higher in tamoxifen-resistant tumors than in primary non-resistant tumors. Subsequent in vitro studies corroborated these results, i.e., after treating sensitive cells with tamoxifen for 1 week, HOTAIR expression levels were found to increase, whereas GREB1 (an ER-induced gene) expression levels were found to decrease. The authors also reported that estradiol-treated MCF7 cells showed decreased HOTAIR and increased GREB1 expression levels, and that after hormone deprivation the HOTAIR and GREB1 levels were restored to their normal levels. In addition, the authors found an ER-binding site near the HOTAIR transcription start site (TSS) and confirmed that estrogen stimulation led to increased ER binding to this site. Finally, the authors showed that HOTAIR can interact with ER and, by doing so, enhance its transcriptional activity and promote tamoxifen-resistant breast cancer progression.

BCAR4 has been reported to act as an oncogenic lncRNA that is involved in the progression of several cancers, including breast cancer [77]. Godinho et al. [78] found that BCAR4 expression in breast cancer is associated with aggressiveness and tamoxifen resistance through targeting the HER2 signaling pathway. The authors suggested that BCAR4 may serve as a suitable target for patients with tamoxifen-resistant breast cancer [79]. In addition, it has been reported that BCAR4 expression may be used to identify a subset of ER-positive and HER2-low expressing breast cancer patients with a poor prognosis who may benefit from anti-HER2/anti-estrogen combination therapy [80].

Another lncRNA, CCAT2, that was previously found to be highly expressed in microsatellite-stable colorectal cancers and to promote their growth, chromosomal instability and metastasis [81], has more recently also been found to be implicated in tamoxifen resistance in breast cancer. Cai et al. [82] found that CCAT2 is overexpressed in tamoxifen-resistant breast cancer cells compared to sensitive cells. In addition, they found that CCAT2 expression knockdown led to an increased apoptosis and an enhanced radio-sensitivity of tamoxifen-resistant breast cancer cells compared to their parental sensitive cells. From their data, they also concluded that the ERK/MAPK signaling pathway may be involved in CCAT2-mediated tamoxifen resistance in breast cancer cells. Others have reported that the expression of eleven other lncRNAs (i.e., PINK1.AS, RP11.259 N19.1, KLF3.AS1, LINC00339, LINC00472, RP11.351I21.11, KB.1460A1.5, PKD1P6.NPIPP1, PDCD4.AS1, KLF3.AS1 PP14571 and RP11.69E11.4) may predict recurrence in ER-positive breast cancer patients treated with tamoxifen [83].

### LncRNAs and targeted therapies

Targeted therapies, also called ‘biologic’ therapies, are designed to act on cancer cells that exhibit specific features. These therapies are generally less likely to harm healthy cells than chemo- and/or radiotherapy. For targeted therapies monoclonal antibodies or small molecule inhibitors are employed that can bind in a specific manner to their target. Trastuzumab and lapatinib, and more recently pertuzumab, are widely used for the treatment of HER2-positive breast cancers. Although these therapies are quite specific, a subset of the patients may develop de novo or acquired resistance. The role of lncRNAs in this resistance appears to be of interest. Cheng et al. [84] found, for instance, that lncRNA UCA1 caused a non-T790 M mutation-associated acquired resistance to epidermal growth factor receptor tyrosine kinase inhibitors (EGFR-TKIs) by activating the AKT/mTOR pathway in non-small cell lung cancer cells. In a similar manner, it has been reported that the UCA1/miR-18/YAP1 axis may regulate trastuzumab resistance in breast cancer cells [85].

Consistent with these results, Dong et al. [86] found that overexpression of lncRNA GAS5 may enhance the sensitivity of gefitinib-resistant lung cancer cells to EGFR-TKIs, at least in part by downregulating IGF-1R. In breast cancer cells, Li et al. [87] found that decreasing the expression of GAS5 led to trastuzumab resistance. Microarray-based analyses revealed that the expression of GAS5 was downregulated in trastuzumab resistant breast cancer cells as well as in trastuzumab-treated primary breast cancer tissues. In addition, it was found that low GAS5 expression levels correlated with a poor prognosis, and that GAS5 expression knockdown in breast cancer-derived SKBR-3 cells led to an increased proliferation. These results were confirmed in vivo through subcutaneous inoculation of SKBR-3 cells transfected with a GAS5 siRNA into nude mice. It was found that tumors in the GAS5 knockdown group grew more rapidly than those in the control group. The authors also found that GAS5 was upregulated after exposure of trastuzumab-resistant cells (SKBR-3/Tr) to lapatinib, which binds to the endogenous PTEN targeting microRNA miR-21. Finally, it was found that lapatinib may inhibit the mTOR signaling pathway in SKBR-3/Tr cells. The authors concluded that GAS5, which is regulated by the mTOR pathway, may act as a competing endogenous RNA to miR-21 to regulate PTEN during the development of trastuzumab resistance in breast cancer cells.

Additionally, it has been found that deregulation of lncRNA ATB may contribute to cancer cell proliferation, migration and invasion. This lncRNA is aberrantly expressed in several cancers, including hepatocellular and colorectal cancers. ATB has been found to be involved in tumor progression through competitive binding to miRNAs and, by doing so, to induce epithelial-mesenchymal transition (EMT) [88]. In breast cancer, ATB expression has been found to be increased in trastuzumab-resistant cells. This increased expression has, in turn, been found to promote tumor cell invasion and metastasis through competitive binding of miR-200c, thereby inducing EMT [89].

## Conclusions and perspectives

Currently, the involvement of lncRNAs in disease development, including cancer, has amply been documented, including its translation from basic science to clinical research. A strong association has, for instance, been reported between lncRNA PCA3 overexpression and malignant transformation of prostate epithelial cells [90–92]. This notion has led to an improvement in the sensitivity of prostate cancer diagnostics [93]. Although PCA3 does not seem to have any relevance in breast cancer, other lncRNAs described in this review may be employed for improvement of breast cancer diagnosis and prognosis, as also therapy. These options need, however, to be substantiated in larger and independent breast cancer cohort studies.

Although it has been found that concurrent targeted and conventional therapeutic regimens show better clinical outcomes in a considerable proportion of breast cancer patients, certain subsets of patients show only modest benefits. Therefore, optimization of these therapies based on novel molecular parameters is expected to lead to even better results and to contribute to a more personalized precision medicine, providing more effective and prolonged responses and improved survival rates. In addition, technological improvements, including the use of less invasive methods, is expected to contribute to the success of precision medicine in the near future. In this respect, lncRNAs may play an important role since they have been encountered in body fluids and exosomes. It will be imperative to uncover in more detail the key mechanisms related to lncRNA expression, sub-cellular localization, gain or loss of function and molecular interaction. For a successful clinical translation, the identification of driver lncRNAs and their preclinical and clinical relevance in breast cancer will also be necessary.

It is anticipated that the development of specific lncRNA-based cancer therapies will enable their use in clinical practice. For example, overexpression of tumor suppressor lncRNAs in cells lacking these RNAs to reverse its function may be employed. Conversely, small interfering RNAs (siRNAs), antisense oligonucleotides (ASOs), short hairpin RNAs (shRNAs) or other molecules may be used to suppress oncogenic lncRNAs. Through clinical studies, lncRNA BCAR4 has for example been identified as being responsible for resistance to the anti-estrogen tamoxifen in patients with breast cancer [65, 94]. Additional studies have shown that BCAR4 overexpression may be associated with more aggressive tumors and shorter progression-free survival rates [78]. Concordantly, Xing et al. [95] found that targeting BCAR4 in highly metastatic breast cancer mouse models using a locked nucleic acid (LNA)-based ASO strategy led to alterations in cell migration and invasion. As another example, it was found that suppressing HOTAIR-EZH2 activity using a peptide nucleic acid-based approach resulted in re-sensitization of ovarian and breast cancer cells [96]. Theoretically, the CRISPR/Cas9 system may also be used for targeting lncRNAs in the cell nucleus. LncRNAs do, however, not seem to be very susceptible to insertions and/or deletions induced by the CRISPR/Cas9 system [97]. In addition, this system may unintentionally affect overlapping and/or neighboring genes [98]. Therefore, siRNAi or ASO-mediated knockdown assays may be better suited to study the functions of lncRNAs and to revert their activities to normal breast tissue conditions.

Due to their novelty and complexity, to date few studies have tested the efficacy of lncRNAs as therapeutic targets in breast cancer patients. However, various studies increasingly support their use as diagnostic, prognostic and/or predictive biomarkers, which already has prompted the initiation of prospective clinical breast cancer trials. One of these trials is aimed at analyzing the efficacy of chemotherapy according to mRNA-lncRNA signatures for high-risk TNBC patients (www.clinicaltrials.gov; identifier NCT02641847). So, understanding the role of lncRNAs in the development and progression of breast cancer may be instrumental for their use as monitoring tools and therapeutic targets, leading to improved personalized precision breast cancer medicine.
